# 4,5,7-Trimeth­oxy-2-methyl-3-(2,4,5-trimethoxy­phen­yl)-1-[3-(2,4,5-tri­methoxy­phen­yl)pentan-2-yl]indane acetone 0.858-solvate

**DOI:** 10.1107/S1600536809018613

**Published:** 2009-05-23

**Authors:** Chang Liu, Guangyu Xu

**Affiliations:** a College of Chemistry and Chemical Engineering, Hunan Normal University, Changsha 410081, People’s Republic of China

## Abstract

In the title compound, C_36_H_48_O_9_.0.858C_3_H_6_O, the five-membered ring adopts an envelope conformation. The acetone solvent mol­ecule was disordered and was refined over two positions with equal occupancies, giving an overall occupancy of 0.858 (4). There are weak intra­molecular C—H⋯O hydrogen bonds and intermolecular C—H⋯π inter­actions in the structure.

## Related literature

For general background, see: Diaz *et al.* (1993 ▶); Hernandez *et al.* (1993 ▶); Menon & Dandiya (1967 ▶); Belova *et al.* (1985 ▶); Xu *et al.* (2009 ▶). For related structures, see: Lemini *et al.* (1990 ▶).
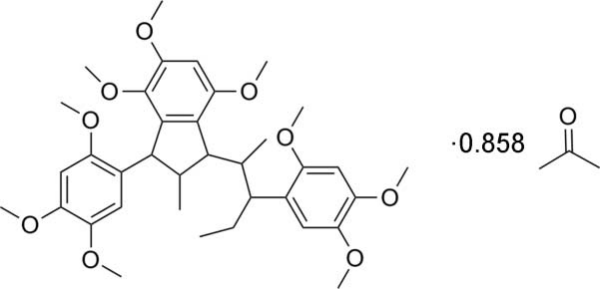


## Experimental

### 

#### Crystal data

C_36_H_48_09·0.858C_3_H_6_O
*M* *_r_* = 674.57
Triclinic, 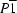

*a* = 8.9234 (10) Å
*b* = 13.2672 (14) Å
*c* = 16.3992 (18) Å
α = 87.757 (2)°
β = 80.0900 (1)°
γ = 76.0220 (1)°
*V* = 1855.9 (4) Å^3^
*Z* = 2
Mo *K*α radiation
μ = 0.09 mm^−1^
*T* = 298 K
0.49 × 0.41 × 0.03 mm

#### Data collection

Bruker SMART APEX area-detector diffractometer
Absorption correction: multi-scan (*SADABS*; Bruker, 1998 ▶) *T*_min_ = 0.959, *T*_max_ = 0.997
9730 measured reflections
6438 independent reflections
3660 reflections with *I* > 2σ(*I*)
*R*_int_ = 0.029

#### Refinement

*R*[*F* ^2^ > 2σ(*F* ^2^)] = 0.055
*wR*(*F* ^2^) = 0.174
*S* = 1.01
6438 reflections
495 parameters
84 restraints
H-atom parameters constrained
Δρ_max_ = 0.40 e Å^−3^
Δρ_min_ = −0.24 e Å^−3^

### 

Data collection: *SMART* (Bruker, 1998 ▶); cell refinement: *SAINT* (Bruker, 1998 ▶); data reduction: *SAINT*; program(s) used to solve structure: *SHELXS97* (Sheldrick, 2008 ▶); program(s) used to refine structure: *SHELXL97* (Sheldrick, 2008 ▶); molecular graphics: *SHELXTL* (Sheldrick, 2008 ▶); software used to prepare material for publication: *SHELXTL*.

## Supplementary Material

Crystal structure: contains datablocks I, global. DOI: 10.1107/S1600536809018613/fb2143sup1.cif

Structure factors: contains datablocks I. DOI: 10.1107/S1600536809018613/fb2143Isup2.hkl

Additional supplementary materials:  crystallographic information; 3D view; checkCIF report

## Figures and Tables

**Table 1:** Hydrogen-bond geometry (Å, °)

*D*—H⋯*A*	*D*—H	H⋯*A*	*D*⋯*A*	*D*—H⋯*A*
C17—H17⋯O7	0.98	2.35	2.820 (3)	109
C25—H25*B*⋯O2	0.96	2.27	2.912 (5)	123
C28—H28*A*⋯*Cg*1^i^	0.96	2.93	3.565 (4)	125

Symmetry code: (i) 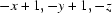. *Cg*1 is the centroid of the C18–C23 ring.

**Fig. 1.**
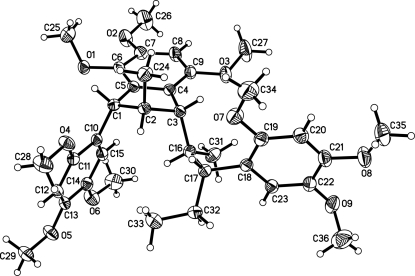
The title molecule without the acetone solvate. The displacement ellipsoids are drawn at the 30% probability level.

**Fig. 2.**
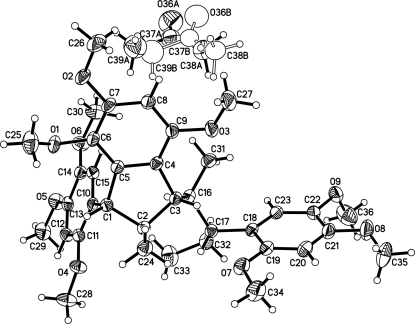
The title molecule with the disordered acetone solvate. The displacement ellipsoids are drawn at the 30% probability level.

**Fig. 3.**
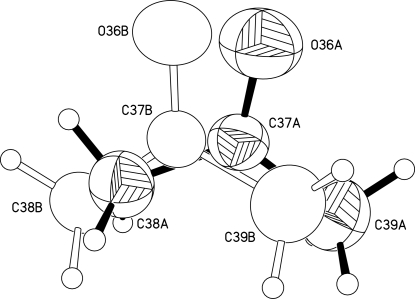
The disordered acetone solvate. The displacement ellipsoids are drawn at the 30% probability level.

**Fig. 4.**
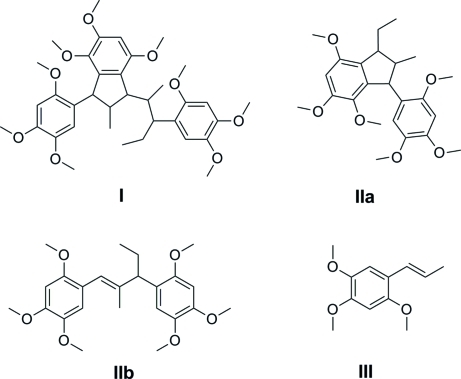
Schematic representations of the title molecule (I) without the acetone solvent olecule, asarone dimers (IIa) and (IIb) (Lemini et al., 1990) and α-asarone monomer (III) (Xu et al., 2009)

## supplementary crystallographic information

### Comment

α-Asarone, (III) (Scheme 2), isolated from the Guatteria guameri plant growing in Southeast Mexico, is reported to be an antiplatelet and hypolipidemic agent (Diaz *et al.*, 1993; Hernandez *et al.*, 1993). In addition, it is known to have sedative, neuroleptic, spasmolytic, antiulcerogenic and antiatherogenic activities (Menon & Dandiya, 1967; Belova *et al.*, 1985). As a part of  our studies on the optimization of the synthesis of α-asarone (Xu *et al.*, 2009), three by-products, two asarone dimers (Lemini *et al.*, 1990) and the asarone trimer, the title compound (I), were isolated and identified from the crude product. The structure of the asarone dimers was reported by Lemini *et al.* (1990). In this paper, the structure of the title compound (I) is reported.

As shown in Fig. 1 and Fig. 2, the five-membered ring C4\C3\C5\C1\C2 has an envelope conformation. C4\C3\C5\C1 is nearly planar with the mean deviation of 0.0043 (3) Å and C2 is situated 0.500 (4) Å out of the C4\C3\C5\C1 plane. The benzene ring (C4 to C9) is almost perpendicular to the other two benzene rings (C10 to C15; C18 to C23) with the interplanar angles of 85.05 (9) and 77.58 (7)°, respectively, while the interplanar angle between the benzene rings (C10 to C15 and C18 to C23) equals to 61.31 (10)°. As shown in Fig. 3, the acetone solvate was disordered and it was refined in two positions with equal occupancies giving the overall occupancy 0.858 (4). This means that the content of acetone is lesser than that of asarone trimer, or in other words, that in some unit cells the acetone molecule is not present. The molecular and crystal structure of the title compound is stabilized by intramolecular weak C-H···O hydrogen bonds and C-H···π-ring electron interactions (Table 1).

### Experimental

In the α-asarone preparation from 2,4,5-trimethoxybenzaldehyde (Xu *et al.*, 2009), the crude product, containing α-asarone, asarone dimers and asarone trimer and other unknown impurities, was dissolved in hot EtOH/H_2_O (V:V 7:3), and then cooled and filtrated. The yellow powder, obtained by concentrating of the filtrate in *vacuo*, was dissolved again in  EtOH/H_2_O (V:V 7:3), and then cooled and filtrated. The filtrate afforded a yellow oil after removal of the solvents under reduced pressure. Fifty grams of the yellow oil was subjected to column chromatography on silica gel and eluted with hexane - ethyl acetate (4:1), the Rf 0.32 fraction was collected and evaporated under vacuum. The residue was crystallized from ethanol to afford the title compound (I). White solid, m.p. 408 K, ^1^H NMR (CDCl_3_, p.p.m.): 0.58 (t, 3 H, J = 9.6 Hz), 0.63 (d, 3 H, J = 9.6 Hz), 1.66 (d, 3 H, J = 8.8 Hz), 1.43 (m, 1 H), 1.73 (m, 2 H,), 2.21 (m, 1 H), 2.83 (dd, 1 H, J = 5.6, 7.2 Hz), 3.20 (m, 1 H), 3.44 (s, 3 H), 3.61 (s, 3 H), 3.73 (s, 3 H), 3.77 (s, 3 H), 3.79 (s, 3 H), 3.86 (s, 3 H), 3.86 (s, 3 H), 3.88 (s, 6 H), 4.21 (d, 1 H, J =5.2 Hz), 6.40 (s, 1 H), 6.43 (s, 1 H), 6.48 (s, 1 H), 6.55 (s, 1 H), 6.59 (s, 1 H). ^13^C NMR (CDCl_3_, p.p.m.): 12.4, 14.9, 21.0, 26.2, 42.1, 49.3, 49.6, 53.1, 55.1, 56.0, 56.2, 56.4, 56.5, 56.5, 56.6, 56.7, 60.0, 96.5, 97.7, 97.9, 113.0, 125.2, 126.7, 127.0, 139.7, 139.9, 142.5, 142.8, 147.0, 147.5, 151.2, 151.8, 152.4, 152.8. The single crystals were obtained by slow evaporation of the title compound dissolved in acetone at room temperature on the third day.

### Refinement

All the H atoms were placed into the calculated idealized positions, with C—H = 0.98 (methine), 0.97 (methylene), 0.96 (methyl) and 0.93 Å (aryl), and were treated in riding mode approximation. (The methyl groups were checked in the difference electron density maps and allowed to rotate freely about their axes.) *U*_iso_(H)=1.5*U*_eq_C_methyl_ or *U*_iso_(H)=1.3*U*_eq_C_methylene_/C_methine_/_aryl_. The acetone solvate appeared to be disordered, and it was refined in two positions. Its occupancy was also refined with assumed equal occupancy at each position because of the proximity of both disordered parts. The following restraints for the disordered acetones have been used: C=O distance was restrained to 1.207 (2) Å; the distances between the neighbour carbons were restrained to 1.344 (2) Å. The displacement parameters of the corresponding atoms were restrained by SIMU 0.05 0.05 for the pairs of the atoms C38A C38B; C39A C39B; O36A O36B; C37A C37B. Moreover the command ISOR 0.05 0.05 was applied for C37A C37B; O36A O36B; C38A C38B C39A C39B (SHELXL-97 (Sheldrick, 2008)).

The refinement under assumption of stoichiometric ratio of both constituing molecules, *i.e.* with occupancy equal to 0.5 in each position, gave worse result: _refine_ls_R_factor_all = 0.1087; _refine_ls_R_factor_gt = 0.0582; _refine_ls_wR_factor_ref = 0.1905; _refine_ls_wR_factor_gt = 0.1492; _refine_ls_goodness_of_fit_ref = 1.019; _refine_ls_restrained_S_all = 1.037. Therefore the non-stioichiometric content of the acetone molecule was given the preference.

### Crystal data

**Table:**

C_36_H_48_09·0.858C_3_H_6_O	*Z* = 2
*M**_r_* = 674.57	*F*(000) = 727
Triclinic, *P*1	*D*_x_ = 1.207 Mg m^−^^3^
Hall symbol: -P 1	Melting point: 408 K
*a* = 8.9234 (10) Å	Mo *K*α radiation, λ = 0.71073 Å
*b* = 13.2672 (14) Å	Cell parameters from 2731 reflections
*c* = 16.3992 (18) Å	θ = 2.5–27.8°
α = 87.757 (2)°	µ = 0.09 mm^−^^1^
β = 80.0900 (1)°	*T* = 298 K
γ = 76.0220 (1)°	Plate, colourless
*V* = 1855.9 (4) Å^3^	0.49 × 0.41 × 0.03 mm

### Data collection

**Table:**

Bruker APEX area-detector diffractometer	6438 independent reflections
Radiation source: fine-focus sealed tube	3660 reflections with *I* > 2σ(*I*)
graphite	*R*_int_ = 0.029
φ and ω scans	θ_max_ = 25.0°, θ_min_ = 1.6°
Absorption correction: multi-scan (*SADABS*; Bruker, 1998)	*h* = −10→10
*T*_min_ = 0.959, *T*_max_ = 0.997	*k* = −15→12
9730 measured reflections	*l* = −19→19

### Refinement

**Table:**

Refinement on *F*^2^	Primary atom site location: structure-invariant direct methods
Least-squares matrix: full	Secondary atom site location: difference Fourier map
*R*[*F*^2^ > 2σ(*F*^2^)] = 0.055	Hydrogen site location: inferred from neighbouring sites
*wR*(*F*^2^) = 0.174	H-atom parameters constrained
*S* = 1.01	*w* = 1/[σ^2^(*F*_o_^2^) + (0.0748*P*)^2^ + 0.7819*P*] where *P* = (*F*_o_^2^ + 2*F*_c_^2^)/3
6438 reflections	(Δ/σ)_max_ < 0.001
495 parameters	Δρ_max_ = 0.40 e Å^−^^3^
84 restraints	Δρ_min_ = −0.24 e Å^−^^3^

### Special details

**Table:**

Geometry. All esds (except the esd in the dihedral angle between two l.s. planes) are estimated using the full covariance matrix. The cell esds are taken into account individually in the estimation of esds in distances, angles and torsion angles; correlations between esds in cell parameters are only used when they are defined by crystal symmetry. An approximate (isotropic) treatment of cell esds is used for estimating esds involving l.s. planes.
Refinement. Refinement of F^2^ against ALL reflections. The weighted R-factor wR and goodness of fit S are based on F^2^, conventional R-factors R are based on F, with F set to zero for negative F^2^. The threshold expression of F^2^ > 2sigma(F^2^) is used only for calculating R-factors(gt) etc. and is not relevant to the choice of reflections for refinement. R-factors based on F^2^ are statistically about twice as large as those based on F, and R- factors based on ALL data will be even larger.

### Fractional atomic coordinates and isotropic or equivalent isotropic displacement parameters (Å^2^)

**Table:**

	*x*	*y*	*z*	*U*_iso_*/*U*_eq_	Occ. (<1)
O1	1.1931 (2)	0.14498 (15)	0.22957 (13)	0.0519 (6)	
O4	0.9453 (3)	0.25903 (16)	−0.01442 (11)	0.0510 (6)	
O2	1.1952 (3)	0.16737 (17)	0.39503 (14)	0.0673 (7)	
O6	0.7166 (3)	−0.01714 (17)	0.20417 (13)	0.0619 (6)	
O5	0.7116 (3)	−0.03625 (16)	0.04828 (13)	0.0601 (6)	
O9	0.0148 (3)	0.66946 (18)	0.36065 (14)	0.0633 (6)	
O7	0.5805 (3)	0.66492 (16)	0.15812 (14)	0.0580 (6)	
O3	0.7466 (3)	0.46292 (17)	0.40510 (13)	0.0604 (6)	
O8	0.1064 (3)	0.84904 (18)	0.33474 (16)	0.0762 (8)	
C10	0.8901 (3)	0.2026 (2)	0.12217 (16)	0.0359 (7)	
C11	0.8868 (3)	0.1895 (2)	0.03872 (17)	0.0386 (7)	
C2	0.8520 (3)	0.4007 (2)	0.13985 (17)	0.0379 (7)	
H2	0.7865	0.4008	0.0976	0.045*	
C4	0.8538 (3)	0.3652 (2)	0.28242 (17)	0.0380 (7)	
C6	1.0852 (3)	0.2234 (2)	0.27625 (18)	0.0420 (7)	
C18	0.3909 (3)	0.5759 (2)	0.22003 (17)	0.0386 (7)	
C14	0.7756 (3)	0.0537 (2)	0.15255 (18)	0.0425 (7)	
C5	0.9700 (3)	0.2873 (2)	0.23899 (17)	0.0371 (7)	
C17	0.4851 (3)	0.4764 (2)	0.17502 (17)	0.0396 (7)	
H17	0.5580	0.4977	0.1297	0.047*	
C3	0.7474 (3)	0.4266 (2)	0.22550 (16)	0.0370 (7)	
H3	0.7293	0.5009	0.2368	0.044*	
C19	0.4390 (4)	0.6688 (2)	0.20851 (19)	0.0449 (7)	
C16	0.5879 (3)	0.3984 (2)	0.22794 (17)	0.0388 (7)	
H16	0.6112	0.3307	0.2006	0.047*	
C9	0.8590 (4)	0.3818 (2)	0.36492 (18)	0.0454 (8)	
C13	0.7717 (3)	0.0435 (2)	0.06860 (18)	0.0430 (7)	
C8	0.9742 (4)	0.3178 (2)	0.40316 (19)	0.0516 (8)	
H8	0.9774	0.3290	0.4584	0.062*	
C22	0.1547 (4)	0.6702 (2)	0.30812 (18)	0.0471 (8)	
C23	0.2479 (3)	0.5802 (2)	0.27077 (17)	0.0422 (7)	
H23	0.2134	0.5193	0.2800	0.051*	
C1	0.9554 (3)	0.2895 (2)	0.14877 (16)	0.0369 (7)	
H1	1.0592	0.2851	0.1156	0.044*	
C7	1.0842 (4)	0.2374 (2)	0.36009 (19)	0.0487 (8)	
C24	0.9528 (4)	0.4779 (2)	0.1168 (2)	0.0527 (8)	
H24A	1.0092	0.4823	0.1609	0.079*	
H24B	0.8873	0.5449	0.1080	0.079*	
H24C	1.0257	0.4553	0.0671	0.079*	
C12	0.8280 (4)	0.1111 (2)	0.01227 (18)	0.0437 (7)	
H12	0.8265	0.1040	−0.0438	0.052*	
C32	0.3788 (4)	0.4259 (3)	0.1338 (2)	0.0533 (8)	
H32A	0.3224	0.3889	0.1757	0.064*	
H32B	0.3021	0.4804	0.1122	0.064*	
C21	0.2052 (4)	0.7620 (2)	0.2949 (2)	0.0539 (9)	
C28	0.9350 (5)	0.2536 (3)	−0.09955 (19)	0.0660 (10)	
H28A	0.8274	0.2628	−0.1055	0.099*	
H28B	0.9942	0.1871	−0.1214	0.099*	
H28C	0.9766	0.3074	−0.1293	0.099*	
C31	0.5048 (4)	0.3847 (3)	0.31565 (19)	0.0545 (9)	
H31A	0.5683	0.3284	0.3421	0.082*	
H31B	0.4057	0.3694	0.3133	0.082*	
H31C	0.4880	0.4475	0.3466	0.082*	
C20	0.3467 (4)	0.7606 (2)	0.2450 (2)	0.0529 (8)	
H20	0.3805	0.8218	0.2357	0.063*	
C29	0.7102 (4)	−0.0519 (3)	−0.0364 (2)	0.0648 (10)	
H29A	0.6718	−0.1123	−0.0424	0.097*	
H29B	0.8147	−0.0616	−0.0669	0.097*	
H29C	0.6433	0.0077	−0.0574	0.097*	
C34	0.6242 (5)	0.7593 (3)	0.1351 (3)	0.0792 (12)	
H34A	0.6383	0.7922	0.1833	0.119*	
H34B	0.5435	0.8044	0.1100	0.119*	
H34C	0.7205	0.7450	0.0963	0.119*	
C27	0.7729 (6)	0.4980 (3)	0.4807 (2)	0.0951 (15)	
H27A	0.6969	0.5615	0.4973	0.143*	
H27B	0.8762	0.5100	0.4735	0.143*	
H27C	0.7633	0.4463	0.5227	0.143*	
C33	0.4646 (5)	0.3512 (3)	0.0641 (2)	0.0806 (12)	
H33A	0.3901	0.3262	0.0397	0.121*	
H33B	0.5346	0.2937	0.0857	0.121*	
H33C	0.5235	0.3864	0.0229	0.121*	
C25	1.3527 (4)	0.1453 (3)	0.2235 (3)	0.0806 (12)	
H25A	1.4164	0.0842	0.1943	0.121*	
H25B	1.3781	0.1457	0.2780	0.121*	
H25C	1.3722	0.2060	0.1941	0.121*	
C30	0.7076 (5)	−0.0056 (3)	0.2900 (2)	0.0758 (11)	
H30A	0.6516	−0.0532	0.3191	0.114*	
H30B	0.6535	0.0642	0.3062	0.114*	
H30C	0.8114	−0.0201	0.3032	0.114*	
C35	0.1433 (6)	0.9472 (3)	0.3104 (3)	0.0986 (15)	
H35A	0.0658	1.0025	0.3402	0.148*	
H35B	0.1442	0.9571	0.2521	0.148*	
H35C	0.2446	0.9473	0.3229	0.148*	
C26	1.2133 (6)	0.1858 (3)	0.4760 (2)	0.0931 (15)	
H26A	1.2986	0.1333	0.4912	0.140*	
H26B	1.1187	0.1839	0.5134	0.140*	
H26C	1.2352	0.2528	0.4788	0.140*	
C36	−0.1154 (5)	0.6869 (4)	0.3199 (3)	0.1014 (15)	
H36A	−0.2094	0.6948	0.3601	0.152*	
H36B	−0.1055	0.6290	0.2844	0.152*	
H36C	−0.1202	0.7490	0.2873	0.152*	
C15	0.8340 (3)	0.1327 (2)	0.17745 (18)	0.0412 (7)	
H15	0.8360	0.1394	0.2335	0.049*	
C38A	0.5792 (18)	0.2552 (10)	0.5382 (9)	0.099 (3)	0.429 (3)
H38A	0.5361	0.2849	0.4905	0.148*	0.429 (3)
H38B	0.6648	0.2845	0.5447	0.148*	0.429 (3)
H38C	0.4998	0.2700	0.5866	0.148*	0.429 (3)
C39A	0.7892 (15)	0.1035 (12)	0.4803 (9)	0.123 (4)	0.429 (3)
H39A	0.7878	0.1183	0.4226	0.184*	0.429 (3)
H39B	0.8182	0.0297	0.4881	0.184*	0.429 (3)
H39C	0.8637	0.1347	0.4989	0.184*	0.429 (3)
O36A	0.5576 (11)	0.0861 (8)	0.5564 (6)	0.128 (3)	0.429 (3)
C37A	0.6341 (13)	0.1455 (9)	0.5276 (8)	0.080 (3)	0.429 (3)
C38B	0.5848 (16)	0.3015 (8)	0.5510 (8)	0.089 (3)	0.429 (3)
H38D	0.5885	0.3306	0.4963	0.133*	0.429 (3)
H38E	0.6729	0.3104	0.5738	0.133*	0.429 (3)
H38F	0.4896	0.3363	0.5855	0.133*	0.429 (3)
C39B	0.7096 (19)	0.1325 (13)	0.4835 (10)	0.130 (4)	0.429 (3)
H39D	0.7284	0.1757	0.4364	0.196*	0.429 (3)
H39E	0.6744	0.0747	0.4671	0.196*	0.429 (3)
H39F	0.8048	0.1074	0.5055	0.196*	0.429 (3)
O36B	0.5069 (10)	0.1506 (7)	0.5948 (5)	0.112 (2)	0.429 (3)
C37B	0.5899 (15)	0.1928 (9)	0.5469 (7)	0.073 (3)	0.429 (3)

### Atomic displacement parameters (Å^2^)

**Table:**

	*U*^11^	*U*^22^	*U*^33^	*U*^12^	*U*^13^	*U*^23^
O1	0.0451 (13)	0.0443 (12)	0.0616 (14)	0.0069 (10)	−0.0179 (11)	−0.0153 (10)
O4	0.0677 (15)	0.0575 (13)	0.0348 (11)	−0.0266 (11)	−0.0111 (10)	0.0008 (10)
O2	0.0777 (17)	0.0596 (14)	0.0572 (14)	0.0192 (12)	−0.0394 (13)	−0.0097 (11)
O6	0.0840 (18)	0.0537 (14)	0.0529 (14)	−0.0287 (13)	−0.0082 (12)	0.0035 (11)
O5	0.0794 (17)	0.0522 (14)	0.0570 (14)	−0.0287 (12)	−0.0132 (12)	−0.0112 (11)
O9	0.0528 (15)	0.0731 (16)	0.0535 (14)	−0.0001 (12)	0.0002 (12)	−0.0050 (11)
O7	0.0507 (14)	0.0446 (13)	0.0778 (16)	−0.0155 (11)	−0.0045 (12)	0.0095 (11)
O3	0.0615 (15)	0.0623 (14)	0.0477 (13)	0.0129 (12)	−0.0179 (11)	−0.0195 (11)
O8	0.0833 (19)	0.0445 (14)	0.0900 (19)	0.0004 (13)	−0.0031 (15)	−0.0185 (13)
C10	0.0336 (16)	0.0352 (15)	0.0363 (16)	0.0001 (13)	−0.0094 (13)	−0.0045 (12)
C11	0.0367 (17)	0.0405 (17)	0.0378 (16)	−0.0071 (13)	−0.0063 (13)	−0.0029 (13)
C2	0.0355 (16)	0.0386 (16)	0.0400 (16)	−0.0059 (13)	−0.0115 (13)	−0.0015 (12)
C4	0.0374 (17)	0.0356 (16)	0.0417 (17)	−0.0054 (13)	−0.0132 (13)	−0.0013 (12)
C6	0.0410 (18)	0.0361 (16)	0.0477 (18)	−0.0001 (14)	−0.0151 (14)	−0.0082 (13)
C18	0.0363 (17)	0.0353 (16)	0.0441 (17)	−0.0041 (13)	−0.0130 (14)	0.0003 (13)
C14	0.0429 (18)	0.0354 (16)	0.0464 (18)	−0.0060 (14)	−0.0045 (14)	−0.0002 (13)
C5	0.0379 (17)	0.0352 (15)	0.0401 (16)	−0.0074 (13)	−0.0127 (13)	−0.0029 (12)
C17	0.0364 (17)	0.0402 (16)	0.0418 (17)	−0.0062 (13)	−0.0096 (13)	−0.0010 (13)
C3	0.0382 (17)	0.0336 (15)	0.0392 (16)	−0.0046 (13)	−0.0115 (13)	−0.0032 (12)
C19	0.0442 (19)	0.0400 (18)	0.0505 (19)	−0.0064 (15)	−0.0142 (15)	0.0027 (14)
C16	0.0373 (17)	0.0349 (15)	0.0441 (17)	−0.0058 (13)	−0.0100 (13)	−0.0011 (12)
C9	0.0439 (19)	0.0457 (18)	0.0437 (18)	−0.0001 (15)	−0.0122 (15)	−0.0091 (14)
C13	0.0429 (18)	0.0370 (17)	0.0478 (18)	−0.0055 (14)	−0.0069 (14)	−0.0124 (14)
C8	0.059 (2)	0.0508 (19)	0.0417 (17)	0.0031 (16)	−0.0212 (16)	−0.0084 (14)
C22	0.046 (2)	0.0473 (19)	0.0439 (18)	−0.0039 (15)	−0.0071 (15)	−0.0023 (14)
C23	0.0442 (18)	0.0386 (17)	0.0435 (17)	−0.0078 (14)	−0.0101 (15)	0.0016 (13)
C1	0.0331 (16)	0.0403 (16)	0.0373 (16)	−0.0069 (13)	−0.0087 (13)	−0.0024 (12)
C7	0.050 (2)	0.0436 (18)	0.0519 (19)	0.0031 (15)	−0.0271 (16)	−0.0030 (14)
C24	0.049 (2)	0.0476 (19)	0.060 (2)	−0.0124 (16)	−0.0070 (16)	0.0039 (15)
C12	0.0497 (19)	0.0449 (18)	0.0362 (16)	−0.0094 (15)	−0.0075 (14)	−0.0087 (13)
C32	0.0424 (19)	0.058 (2)	0.062 (2)	−0.0067 (16)	−0.0194 (16)	−0.0123 (16)
C21	0.062 (2)	0.0394 (18)	0.056 (2)	−0.0003 (16)	−0.0149 (18)	−0.0066 (15)
C28	0.092 (3)	0.078 (3)	0.0403 (19)	−0.040 (2)	−0.0183 (19)	0.0098 (17)
C31	0.044 (2)	0.058 (2)	0.058 (2)	−0.0082 (16)	−0.0097 (16)	0.0134 (16)
C20	0.057 (2)	0.0379 (18)	0.066 (2)	−0.0120 (16)	−0.0148 (18)	0.0027 (15)
C29	0.074 (3)	0.061 (2)	0.066 (2)	−0.0199 (19)	−0.018 (2)	−0.0195 (18)
C34	0.071 (3)	0.059 (2)	0.110 (3)	−0.029 (2)	−0.005 (2)	0.013 (2)
C27	0.114 (4)	0.090 (3)	0.064 (3)	0.029 (3)	−0.034 (3)	−0.038 (2)
C33	0.071 (3)	0.093 (3)	0.081 (3)	−0.014 (2)	−0.023 (2)	−0.034 (2)
C25	0.050 (2)	0.088 (3)	0.099 (3)	−0.002 (2)	−0.013 (2)	−0.025 (2)
C30	0.092 (3)	0.086 (3)	0.057 (2)	−0.038 (2)	−0.014 (2)	0.017 (2)
C35	0.101 (4)	0.042 (2)	0.145 (4)	0.001 (2)	−0.019 (3)	−0.018 (2)
C26	0.113 (4)	0.088 (3)	0.071 (3)	0.022 (3)	−0.058 (3)	−0.011 (2)
C36	0.064 (3)	0.151 (5)	0.086 (3)	−0.028 (3)	−0.001 (3)	−0.012 (3)
C15	0.0439 (18)	0.0403 (17)	0.0370 (16)	−0.0046 (14)	−0.0071 (14)	−0.0040 (13)
C38A	0.104 (4)	0.099 (5)	0.092 (4)	−0.017 (4)	−0.024 (3)	0.003 (4)
C39A	0.121 (6)	0.128 (5)	0.119 (5)	−0.027 (4)	−0.021 (4)	−0.005 (4)
O36A	0.113 (4)	0.130 (4)	0.142 (4)	−0.040 (4)	−0.018 (3)	0.024 (3)
C37A	0.078 (4)	0.081 (4)	0.077 (4)	−0.017 (4)	−0.013 (3)	0.011 (4)
C38B	0.096 (4)	0.090 (5)	0.085 (4)	−0.024 (4)	−0.026 (3)	0.005 (4)
C39B	0.132 (6)	0.132 (5)	0.124 (5)	−0.025 (4)	−0.021 (4)	−0.004 (4)
O36B	0.104 (4)	0.120 (4)	0.122 (4)	−0.053 (3)	−0.018 (3)	0.010 (3)
C37B	0.076 (4)	0.075 (4)	0.071 (4)	−0.019 (4)	−0.018 (3)	0.004 (4)

### Geometric parameters (Å, °)

**Table:**

O1—C6	1.386 (3)	C32—C33	1.515 (5)
O1—C25	1.411 (4)	C32—H32A	0.9700
O4—C11	1.377 (3)	C32—H32B	0.9700
O4—C28	1.420 (3)	C21—C20	1.378 (5)
O2—C7	1.372 (3)	C28—H28A	0.9600
O2—C26	1.403 (4)	C28—H28B	0.9600
O6—C14	1.379 (3)	C28—H28C	0.9600
O6—C30	1.408 (4)	C31—H31A	0.9600
O5—C13	1.371 (3)	C31—H31B	0.9600
O5—C29	1.415 (4)	C31—H31C	0.9600
O9—C22	1.391 (4)	C20—H20	0.9300
O9—C36	1.406 (5)	C29—H29A	0.9600
O7—C19	1.376 (4)	C29—H29B	0.9600
O7—C34	1.418 (4)	C29—H29C	0.9600
O3—C9	1.380 (3)	C34—H34A	0.9600
O3—C27	1.417 (4)	C34—H34B	0.9600
O8—C21	1.380 (4)	C34—H34C	0.9600
O8—C35	1.444 (5)	C27—H27A	0.9600
C10—C15	1.391 (4)	C27—H27B	0.9600
C10—C11	1.393 (4)	C27—H27C	0.9600
C10—C1	1.520 (4)	C33—H33A	0.9600
C11—C12	1.387 (4)	C33—H33B	0.9600
C2—C24	1.516 (4)	C33—H33C	0.9600
C2—C3	1.547 (4)	C25—H25A	0.9600
C2—C1	1.557 (4)	C25—H25B	0.9600
C2—H2	0.9800	C25—H25C	0.9600
C4—C9	1.389 (4)	C30—H30A	0.9600
C4—C5	1.391 (4)	C30—H30B	0.9600
C4—C3	1.519 (4)	C30—H30C	0.9600
C6—C5	1.380 (4)	C35—H35A	0.9600
C6—C7	1.393 (4)	C35—H35B	0.9600
C18—C23	1.388 (4)	C35—H35C	0.9600
C18—C19	1.396 (4)	C26—H26A	0.9600
C18—C17	1.523 (4)	C26—H26B	0.9600
C14—C15	1.381 (4)	C26—H26C	0.9600
C14—C13	1.396 (4)	C36—H36A	0.9600
C5—C1	1.506 (4)	C36—H36B	0.9600
C17—C32	1.538 (4)	C36—H36C	0.9600
C17—C16	1.553 (4)	C15—H15	0.9300
C17—H17	0.9800	C38A—C37A	1.426 (11)
C3—C16	1.550 (4)	C38A—H38A	0.9600
C3—H3	0.9800	C38A—H38B	0.9600
C19—C20	1.387 (4)	C38A—H38C	0.9600
C16—C31	1.527 (4)	C39A—C37A	1.456 (12)
C16—H16	0.9800	C39A—H39A	0.9600
C9—C8	1.389 (4)	C39A—H39B	0.9600
C13—C12	1.379 (4)	C39A—H39C	0.9600
C8—C7	1.383 (4)	O36A—C37A	1.196 (9)
C8—H8	0.9300	C38B—C37B	1.436 (11)
C22—C23	1.377 (4)	C38B—H38D	0.9600
C22—C21	1.394 (4)	C38B—H38E	0.9600
C23—H23	0.9300	C38B—H38F	0.9600
C1—H1	0.9800	C39B—C37B	1.459 (12)
C24—H24A	0.9600	C39B—H39D	0.9600
C24—H24B	0.9600	C39B—H39E	0.9600
C24—H24C	0.9600	C39B—H39F	0.9600
C12—H12	0.9300	O36B—C37B	1.203 (9)
			
C6—O1—C25	116.9 (2)	H32A—C32—H32B	107.6
C11—O4—C28	117.8 (2)	C20—C21—O8	124.7 (3)
C7—O2—C26	118.4 (3)	C20—C21—C22	119.4 (3)
C14—O6—C30	117.7 (2)	O8—C21—C22	115.8 (3)
C13—O5—C29	117.9 (2)	O4—C28—H28A	109.5
C22—O9—C36	113.6 (3)	O4—C28—H28B	109.5
C19—O7—C34	118.8 (3)	H28A—C28—H28B	109.5
C9—O3—C27	117.6 (2)	O4—C28—H28C	109.5
C21—O8—C35	116.2 (3)	H28A—C28—H28C	109.5
C15—C10—C11	117.0 (3)	H28B—C28—H28C	109.5
C15—C10—C1	123.2 (2)	C16—C31—H31A	109.5
C11—C10—C1	119.8 (2)	C16—C31—H31B	109.5
O4—C11—C12	123.2 (2)	H31A—C31—H31B	109.5
O4—C11—C10	115.5 (2)	C16—C31—H31C	109.5
C12—C11—C10	121.3 (3)	H31A—C31—H31C	109.5
C24—C2—C3	111.1 (2)	H31B—C31—H31C	109.5
C24—C2—C1	110.8 (2)	C21—C20—C19	120.5 (3)
C3—C2—C1	105.2 (2)	C21—C20—H20	119.7
C24—C2—H2	109.9	C19—C20—H20	119.7
C3—C2—H2	109.9	O5—C29—H29A	109.5
C1—C2—H2	109.9	O5—C29—H29B	109.5
C9—C4—C5	118.6 (2)	H29A—C29—H29B	109.5
C9—C4—C3	130.2 (3)	O5—C29—H29C	109.5
C5—C4—C3	110.9 (2)	H29A—C29—H29C	109.5
C5—C6—O1	118.6 (2)	H29B—C29—H29C	109.5
C5—C6—C7	118.7 (3)	O7—C34—H34A	109.5
O1—C6—C7	122.7 (2)	O7—C34—H34B	109.5
C23—C18—C19	116.5 (3)	H34A—C34—H34B	109.5
C23—C18—C17	121.5 (3)	O7—C34—H34C	109.5
C19—C18—C17	121.8 (3)	H34A—C34—H34C	109.5
O6—C14—C15	125.5 (3)	H34B—C34—H34C	109.5
O6—C14—C13	115.5 (3)	O3—C27—H27A	109.5
C15—C14—C13	119.0 (3)	O3—C27—H27B	109.5
C6—C5—C4	122.1 (2)	H27A—C27—H27B	109.5
C6—C5—C1	127.1 (3)	O3—C27—H27C	109.5
C4—C5—C1	110.7 (2)	H27A—C27—H27C	109.5
C18—C17—C32	110.8 (2)	H27B—C27—H27C	109.5
C18—C17—C16	114.7 (2)	C32—C33—H33A	109.5
C32—C17—C16	112.6 (2)	C32—C33—H33B	109.5
C18—C17—H17	106.0	H33A—C33—H33B	109.5
C32—C17—H17	106.0	C32—C33—H33C	109.5
C16—C17—H17	106.0	H33A—C33—H33C	109.5
C4—C3—C2	101.2 (2)	H33B—C33—H33C	109.5
C4—C3—C16	116.6 (2)	O1—C25—H25A	109.5
C2—C3—C16	110.8 (2)	O1—C25—H25B	109.5
C4—C3—H3	109.3	H25A—C25—H25B	109.5
C2—C3—H3	109.3	O1—C25—H25C	109.5
C16—C3—H3	109.3	H25A—C25—H25C	109.5
O7—C19—C20	122.0 (3)	H25B—C25—H25C	109.5
O7—C19—C18	116.7 (3)	O6—C30—H30A	109.5
C20—C19—C18	121.3 (3)	O6—C30—H30B	109.5
C31—C16—C3	113.2 (2)	H30A—C30—H30B	109.5
C31—C16—C17	113.7 (2)	O6—C30—H30C	109.5
C3—C16—C17	110.2 (2)	H30A—C30—H30C	109.5
C31—C16—H16	106.4	H30B—C30—H30C	109.5
C3—C16—H16	106.4	O8—C35—H35A	109.5
C17—C16—H16	106.4	O8—C35—H35B	109.5
O3—C9—C8	123.1 (3)	H35A—C35—H35B	109.5
O3—C9—C4	117.1 (2)	O8—C35—H35C	109.5
C8—C9—C4	119.8 (3)	H35A—C35—H35C	109.5
O5—C13—C12	124.5 (3)	H35B—C35—H35C	109.5
O5—C13—C14	115.9 (3)	O2—C26—H26A	109.5
C12—C13—C14	119.6 (3)	O2—C26—H26B	109.5
C7—C8—C9	120.8 (3)	H26A—C26—H26B	109.5
C7—C8—H8	119.6	O2—C26—H26C	109.5
C9—C8—H8	119.6	H26A—C26—H26C	109.5
C23—C22—O9	120.8 (3)	H26B—C26—H26C	109.5
C23—C22—C21	119.0 (3)	O9—C36—H36A	109.5
O9—C22—C21	120.2 (3)	O9—C36—H36B	109.5
C22—C23—C18	123.2 (3)	H36A—C36—H36B	109.5
C22—C23—H23	118.4	O9—C36—H36C	109.5
C18—C23—H23	118.4	H36A—C36—H36C	109.5
C5—C1—C10	114.4 (2)	H36B—C36—H36C	109.5
C5—C1—C2	101.8 (2)	C14—C15—C10	122.7 (3)
C10—C1—C2	114.6 (2)	C14—C15—H15	118.6
C5—C1—H1	108.6	C10—C15—H15	118.6
C10—C1—H1	108.6	O36A—C37A—C38A	122.5 (13)
C2—C1—H1	108.6	O36A—C37A—C39A	118.3 (12)
O2—C7—C8	124.0 (3)	C38A—C37A—C39A	119.2 (11)
O2—C7—C6	116.1 (3)	C37B—C38B—H38D	109.5
C8—C7—C6	119.9 (3)	C37B—C38B—H38E	109.5
C2—C24—H24A	109.5	H38D—C38B—H38E	109.5
C2—C24—H24B	109.5	C37B—C38B—H38F	109.5
H24A—C24—H24B	109.5	H38D—C38B—H38F	109.5
C2—C24—H24C	109.5	H38E—C38B—H38F	109.5
H24A—C24—H24C	109.5	C37B—C39B—H39D	109.5
H24B—C24—H24C	109.5	C37B—C39B—H39E	109.5
C13—C12—C11	120.4 (3)	H39D—C39B—H39E	109.5
C13—C12—H12	119.8	C37B—C39B—H39F	109.5
C11—C12—H12	119.8	H39D—C39B—H39F	109.5
C33—C32—C17	114.5 (3)	H39E—C39B—H39F	109.5
C33—C32—H32A	108.6	O36B—C37B—C38B	123.2 (12)
C17—C32—H32A	108.6	O36B—C37B—C39B	120.3 (13)
C33—C32—H32B	108.6	C38B—C37B—C39B	116.4 (11)
C17—C32—H32B	108.6		

### Hydrogen-bond geometry (Å, °)

**Table:**

*D*—H···*A*	*D*—H	H···*A*	*D*···*A*	*D*—H···*A*
C17—H17···O7	0.98	2.35	2.820 (3)	109
C25—H25B···O2	0.96	2.27	2.912 (5)	123
C28—H28A···Cg1^i^	0.96	2.93	3.565 (4)	125

Symmetry codes: (i) −*x*+1, −*y*+1, −*z*.

## References

[bb1] Belova, L. F., Alibekov, S. D., Baginskaya, A. I., Sokolov, S. Y., Pokrovskaya, G. V., Stikhin, V. A., Trumpe, T. & Gorodnyuk, T. I. (1985). *Farmakol. Toksikol.* **48**, 17–20. 4085624

[bb2] Bruker (1998). *SADABS*, *SMART* and *SAINT* Bruker AXS Inc., Madison, Wisconsin, USA.

[bb3] Diaz, F., Munoz, H., Labarrios, F., Chamorro, G., Salazar, M., Morelos, M. E. & Tamariz, J. (1993). *Med. Chem. Res.* **3**, 101–109.

[bb4] Hernandez, A., Lourdes Lopez, M., Chamorro, G. & Mendoza-Figueroa, T. (1993). *Planta Med.* **59**, 121–124. 10.1055/s-2006-959625 8488190

[bb5] Lemini, C., Mandoki, J. J., Cruz-Almanza, R. & Toscano, R. A. (1990). *Acta Cryst.* C**46**, 1542–1545.

[bb6] Menon, M. K. & Dandiya, P. C. (1967). *J. Pharm. Pharmacol.* **9**, 170–175. 10.1111/j.2042-7158.1967.tb08060.x 4382337

[bb7] Sheldrick, G. M. (2008). *Acta Cryst.* A**64**, 112–122. 10.1107/S0108767307043930 18156677

[bb8] Xu, G., Liu, C., Zhang, W. & Zuo, G. (2009). *Org. Prep. Proced. Int.* **41**, 153–156.

